# A Pupil Segmentation Algorithm Based on Fuzzy Clustering of Distributed Information

**DOI:** 10.3390/s21124209

**Published:** 2021-06-19

**Authors:** Kemeng Bai, Jianzhong Wang, Hongfeng Wang

**Affiliations:** School of Mechatronical Engineering, Beijing Institute of Technology, Beijing 100081, China; 3120170114@bit.edu.cn (K.B.); 3120185177@bit.edu.cn (H.W.)

**Keywords:** pupil detection, image segmentation, fuzzy clustering, local features, head-mounted eye-tracking system

## Abstract

Pupil segmentation is critical for line-of-sight estimation based on the pupil center method. Due to noise and individual differences in human eyes, the quality of eye images often varies, making pupil segmentation difficult. In this paper, we propose a pupil segmentation method based on fuzzy clustering of distributed information, which first preprocesses the original eye image to remove features such as eyebrows and shadows and highlight the pupil area; then the Gaussian model is introduced into global distribution information to enhance the classification fuzzy affiliation for the local neighborhood, and an adaptive local window filter that fuses local spatial and intensity information is proposed to suppress the noise in the image and preserve the edge information of the pupil details. Finally, the intensity histogram of the filtered image is used for fast clustering to obtain the clustering center of the pupil, and this binarization process is used to segment the pupil for the next pupil localization. Experimental results show that the method has high segmentation accuracy, sensitivity, and specificity. It can accurately segment the pupil when there are interference factors such as light spots, light reflection, and contrast difference at the edge of the pupil, which is an important contribution to improving the stability and accuracy of the line-of-sight tracking.

## 1. Introduction

With the continuous development of computer vision and artificial intelligence technology, human-eye-tracking techniques are increasingly used in clinical medicine [1], psychology [2,3], recognition systems [4,5], human–computer interaction [6,7], and other fields. Controlling robot motion through line-of-sight-tracking technology is one of the important development directions of human–computer interaction. The pupil is an important feature of the human eye, and pupil detection is often required in the sight-tracking process to perform sight estimation with the relative motion changes of the pupil. Pupil detection usually includes steps such as eye image preprocessing, pupil feature extraction, and localization, etc. Each step of successfully processing the eye image significantly affects the accuracy of the final line-of-sight estimation [6,7,8,9,10].

Currently, there are many methods for pupil feature extraction, such as the threshold method [4,8,9], region method [10], random field method [11], neural network method [5], and clustering method [12]. These methods segment different types of images to different degrees. The threshold segmentation method and the region segmentation method can segment the corresponding features for high-quality images, but the segmentation accuracy is still relatively low, and the processing of the edge is also relatively poor. The segmentation method of neural network learning needs an extensive training and learning process and has a large amount of computation and a complex model, but the segmentation result is relatively good. The extraction of pupil features seems simple, but it is difficult to achieve accurate pupil segmentation because eye images may be affected by noise, unfavorable illumination, and acquisition conditions. In addition, eye images are complex and include many details, such as eyelids, eyelashes, and so on. At the same time, the poor quality of the pupil characteristics of the eye image, including the invasion of low-contrast objects, the invasion of high-intensity objects, and the low contrast between the pupil and the iris, leads to the missing edge information and inaccurate segmentation of the segmentation target.

Reference [9] improved the Otsu algorithm based on a probabilistic statistical model to obtain the threshold of the pupil region for binarization to segment the pupil. However, the probabilistic model is tedious and statistical in the global scope of the eye image, the obtained pupil threshold is a large range of values, and the segmented pupil often contains other non-pupil regions, for the pupil information characteristics are not clear badly segmented. In terms of the clustering segmentation algorithm mentioned in the literature [12,13,14,15,16,17,18,19], feature extraction segmentation of images has worked well for segmentation of infrared electrical appliances images, brain MRI images, fundus vascular images, and breast density images. Reference [12] used the k-means clustering segmentation algorithm to classify eye images. For pixel classification, there are only hard kernel classifications of belonging and non-belonging, which make it hard to determine the value of k. The classical fuzzy c-means clustering (FCM) algorithm uses the grayscale intensity information of pixels as the feature space and does not contain spatial contextual information for pixel classification, which is very sensitive to image noise and intensity inhomogeneity. Reference [13] proposed an improved FCM algorithm to segment the optical interference fringes, with image noise as correction function to build the objective function, which has good noise resistance and segmentation effects, but there is a relatively small number of characteristics, too many parameters are introduced in the process of solving the objective function, and more values need to be set. Reference [14] proposed an FCM-clustering fundus-blood-vessel segmentation algorithm based on local line-structure constraints, where the line structure fully considers the characteristics of the vessel structure, and the segmented blood vessel structure has good continuity and high detection sensitivity. Reference [15] proposed an enhanced FCM fuzzy clustering algorithm that forms a linear weighted sum in the original image and its local spatial neighborhood, which effectively improves the calculation efficiency of the segmentation process and accelerates the image clustering. Reference [16], based on Reference [15], proposed an adaptive local window filter to distinguish adjacent pixels in the local window with weighting coefficients, and then the intensity histogram of the filtered image is quickly clustered. References [17,18] showed an improved enhanced FCM algorithm based on reliable spatial contextual information to control the influence between local neighboring pixels, effectively balance noise and preserve image details, and improve the robustness and accuracy of the clustering algorithm, but the computational complexity is high. Reference [19] proposed a fuzzy clustering segmentation algorithm based on distribution information for low-contrast infrared power devices, where the IFCM intuitionistic fuzzy algorithm introduces local distribution information and measures the difference between clustering centers and data points, but too many parameters are introduced, and more experience values are needed for parameter values. Reference [20] researched a method based on Dempster–Shafer evidence theory to model and fuse the incomplete information that can cope better with distributed information.

To segment the pupil from the eye image, including the eye images with disturbed pupil features, it is necessary to detect and remove all other components of the eye. This paper proposes several improvements, mainly including the introduction of global distribution information in the form of a Gaussian model, combined with local intensity distribution information, and studies a fuzzy c-means clustering algorithm based on distribution information to more accurately segment the pupil pixels and for accurate pupil localization and sight estimation.

The remainder of this paper is organized as follows: Section 2 presents the proposed method in detail. Section 3 describes the experiments and shows the experimental results. Section 4 concludes the whole work.

## 2. Proposed Method

**Figure 1:**
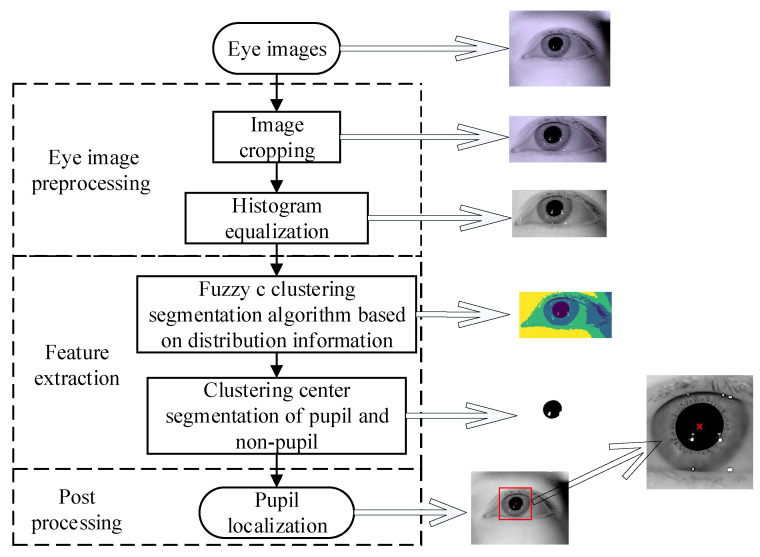
Flow chart of the pupil detection in this article. First, eye image preprocessing, as described in Section 2.2. Second, feature extraction, as described in Section 2.3 and Section 2.4: the fuzzy c clustering algorithm based on distribution information is used to obtain the cluster center of pupils and non-pupils. Then, we compare the size of the clustering center to get pupil cluster center. Finally, postprocessing, as described in Section 2.5: we detect the pupil region to obtain the location of the pupil in eye image.

The algorithm flow chart proposed in this paper is shown in Figure 1. First, the eye image is preprocessed, then the fuzzy c clustering algorithm based on distribution information is used to obtain the cluster centers of pupils and non-pupils; we compare the size of the clustering centers to get the pupil cluster center. Finally, the pupil region is detected to obtain the location of the pupil in the eye image.

### 2.1. Fuzzy C-Means Clustering Algorithm

The standard FCM (fuzzy c-means) algorithm [21] can naturally and non-probabilistically assign each object by using fuzzy affiliation. The objective function *J* is minimized by iteratively updating the affiliation and clustering centers. The objective function is as in Equation (1). Let $X={\left[x_{1},x_{2},\cdots ,x_{N}\right]}^{T}$ denote N pixels in an image to be partitioned into c clusters, which needs to satisfy the condition shown in Equation (2).

(1) $$J=\sum_{j=1}^{N}\sum_{i=1}^{C}u_{ij}^{m}{\left\|x_{j}-v_{i}\right\|}^{2}$$

(2) $$\sum_{i=1}^{C}u_{ij}=1,\;0\leq u_{ij}\leq 1$$

where $u_{ij}$ is the affiliation of the pixel $x_{j}$ in the *i*-th cluster, $v_{i}$ is the cluster center of the *i*-th cluster, ‖🞄‖ is the distance measure, and *m* is the cluster fuzziness. The affiliation $u_{ij}$ and the cluster center $v_{i}$ are calculated iteratively until they reach the optimum.

### 2.2. Eye Image Preprocessing

**Figure 2:**
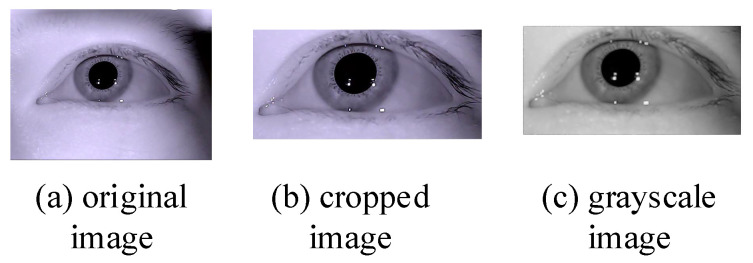
Preprocessing result. (**a**) Original image; (**b**) Cropped image; (**c**) Grayscale image.

A typical eye-tracking-system eye camera captures an eye image much larger than the eye region and also contains non-eye regions such as eyebrows. Therefore, the eye image is cropped to locate the eye region in the preprocessing process [22]. The eye image region of interest obtained after cropping is used as the initial image for subsequent image processing and grayed out; then the grayed-out image is processed using contrast-limited adaptive histogram equalization (CLAHE) to enhance the contrast between the pupil and the non-pupil. The results of the preprocessing process are shown in Figure 2: (a) is the original eye image by the eye camera capturing; (b) is the cropped eye image using Reference [12] method; (c) is the grayscale eye image that enhanced the contrast between pupil and non-pupil region.

### 2.3. Feature Extraction

#### 2.3.1. Feature Extraction Based on Gaussian Model Global Distribution Information

**Figure 3:**
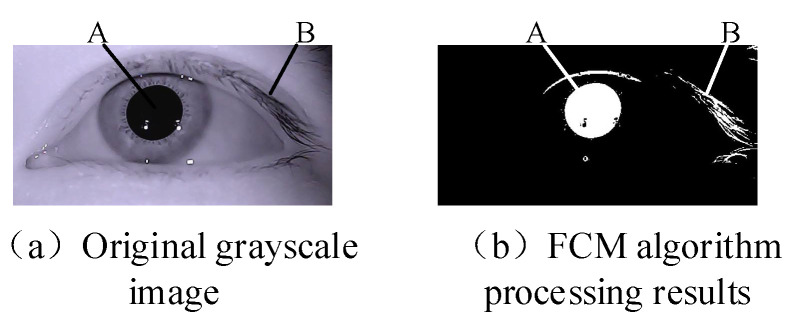
FCM segmentation results. (**a**) Original grayscale image, point A is one pixel point in the pupil region, point B is another pixel point in the eyelashes region, and the two pixel points have very close gray values. Figure 3b is the segmentation result, and point A and point B are the same cluster. (**b**) FCM algorithm processing results.

The histogram of the grayscale distribution of the eye image has a relatively uniform and clear distribution of the intensity of the features in each part of the eye. The traditional fuzzy clustering algorithm often classifies the pixels with the relative intensity similar to the target class. For segmenting the pupil in the eye image, if the global distribution information is lacking, factors such as noise, high gray value, and intensity interference will have a greater impact on the segmentation result. In Equation (1), for each pixel *x* in the image, a larger degree of membership will assign it to a smaller one: ${\left\|x_{j}-v_{i}\right\|}^{2}\left(i=1,\cdots ,c\right)$. However, the distance metric ${\left\|x_{j}-v_{i}\right\|}^{2}$ is only the pixel intensity value, which will lead to errors in the eye diagram classification, since under this metric, the pupil pixels of the eye image are no different from other pixels with similar gray values. As shown in Figure 3, the eyelashes of the pupil point A and the similar gray-value point B are regarded as one category by the FCM algorithm.

**Figure 4:**
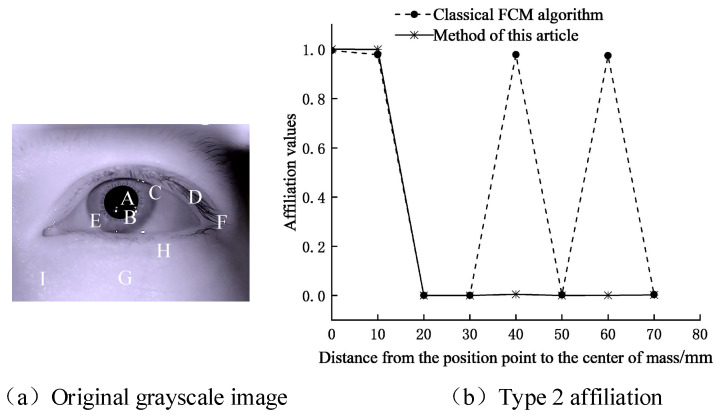
Comparison of the second type of affiliation at different locations. (**a**) Original grayscale image; (**b**) Type 2 affiliation. The second-class affiliation values of point A—point I obtained by the clustering algorithm in this paper, where points A and B are the points in the pupil area, and point C—point I are the points at different distances from the pupil region, and point D and point F in the non-pupil area have close intensity values to point A and point B.

To solve these problems, the global distribution information is introduced into the objective function of the FCM algorithm [19]. When the distance from the possible region of the pupil to the center of mass becomes shorter, the probability of the pixel becoming a target increases. The fluctuation in fuzzy clustering is reflected in the affiliation, and thus the enhanced affiliation $W_{kj}$ is introduced to adapt to the spatial position, which is represented by the Gaussian model, meaning that the closer the pixel is to the centroid, the more likely it is to belong to the target. When the pixel is located at the center of mass, the probability intensity profile peaks, as shown in Equation (3).

(3) $$W_{kj}=\frac{1}{\frac{1}{\sqrt{2\pi }}\exp\left(-\frac{{\left\|l_{j}-l_{k}\right\|}^{2}}{2\sigma^{2}}\right)}$$

where $l_{j}$ is the coordinate of pixel *j*, $l_{k}$ is the coordinate of the center of mass of the possible pupil area, and σ is the variance (σ=3). As shown in Figure 4, comparison of the second type of affiliation at different locations.

Figure 4a provides the original grayscale eye image. Figure 4b shows that due to the lack of spatial information, the affiliation values calculated by the FCM algorithm at different locations are the same. Under low-contrast conditions, the classification without considering the spatial information causes a higher error rate. In this part, we introduce global distribution information to enhance the affiliation, which decreases with the increase of the centroid distance and obtains higher classification accuracy, proving the effectiveness of introducing global distribution information in segmenting the pupil.

#### 2.3.2. Adaptive Feature Extraction of Local Distribution Intensity

We define an adaptive local window filter whose weighting coefficient is determined based on the corresponding local space and gray level with the central pixel. The filtering process consists of two steps: the first step is to evaluate the neighboring pixels of the local window pixels and distinguish unreliable neighbors and reliable neighbors; the second step is to use these reliable neighbors to calculate the new pixel intensity value to produce a filtered image.

The existing pixel reliability assessment for local window, compares the central value of the local 5 × 5 neighborhood with the intensity of 24 neighborhood pixel values in the neighborhood, obtaining the two deviations to assess the pixel reliability. When the appearance of the background and foreground changes consistently, this evaluation method can distinguish between reliable neighboring pixels and unreliable neighboring pixels. However, when the appearance of the background and foreground changes locally, this evaluation method ignores the difference between neighboring pixels in the neighborhood and cannot evaluate the reliability of neighboring pixels well [7]. Meanwhile, the average value of pixels in the local neighborhood is used as the evaluation reference value, which is greatly affected by the extreme values in the neighborhood, and the reliability of the pixels cannot be evaluated well [15,16,18]. Therefore, we use half of the mean and intermediate values of intensity within the local neighborhood as a reference for comparison to assess the pixel reliability. This can cope well with the pixel variations within the local neighborhood to assess the distinction.

**Figure 5:**
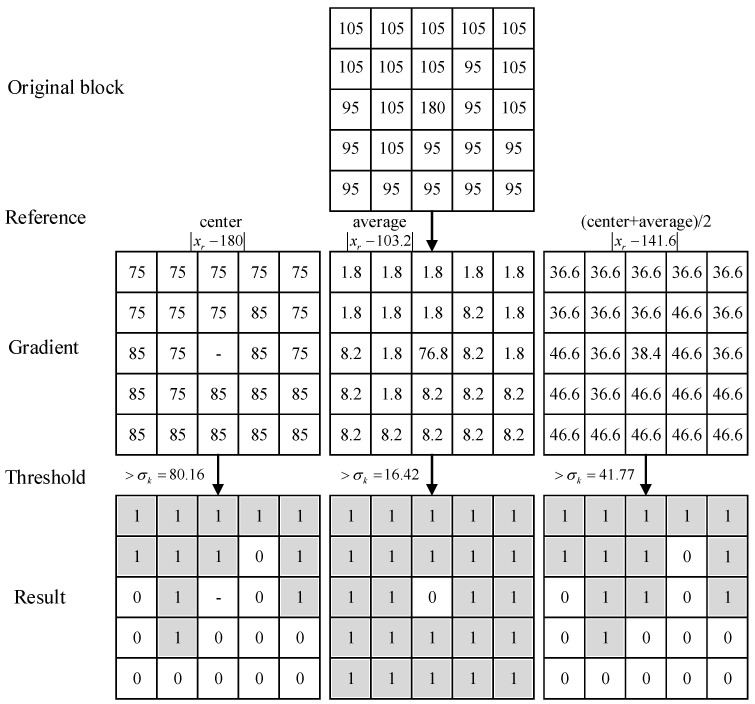
Reliability evaluation of locally distributed neighborhood pixels. The first line represents a local 5 × 5 neighborhood original block; the second line is the reference value for pixel comparison in the neighborhood; the third line provides the pixel values after the contrast difference in the neighborhood block; the fourth line shows the deviation threshold; the fifth line presents the result of reliability evaluation.

The specific filtering process is as follows:

(1) Define a local square window centered on pixel k and set the window size to 5 × 5 throughout the filtering process.

(2) Find the middle pixel value $x_{c}$ and the average intensity value $\bar{x_{k}}$, compare the reference value to $x_{o}=\frac{\left(x_{c}+\bar{x_{k}}\right)}{2}$, and calculate the deviation $\sigma_{k}$ of the intensity value from the reference value in the neighborhood $N_{k}$, as in Equation (4).

(4) $$\sigma_{k}=\sqrt{\sum_{r\in N_{k}}{\left(x_{r}-x_{o}\right)}^{2}/n_{k}}$$

where $x_{r}$ represents the intensity value of the pixel *r* in $N_{k}$, and $n_{k}$ ($n_{k}=25$) is the number of pixels in $N_{k}$. If the difference between $x_{r}$ and $x_{o}$ is greater than $\sigma_{k}$, the pixel *r* is evaluated as unreliable, otherwise it is evaluated as reliable. The local neighborhood pixel reliability is evaluated as shown in Figure 5. The reliability of three different comparison reference values is compared; when the comparison value $>\sigma_{k}$, we regard the pixels as unreliable in the neighborhood and record them as 0, and vice versa otherwise: the other is evaluated as reliable and pixels are recorded as 1.

(3) Define a local window filter and calculate its window weighting coefficient $C_{kr}$, as in Equation (5).

(5) $$C_{kr}=\left\{ \begin{array}{ll} C_{kr\_s}🞄C_{kr\_g}, & if\;r\in N_{r}\\ 0, & otherwise \end{array}\right.$$

where $C_{kr\_s}$ and $C_{kr\_g}$ are the spatial and grayscale terms determined by local spatial distance and local grayscale intensity difference between neighboring pixel *r* and the central pixel $x_{c}$. They are defined as in Equations (6) and (7).

(6) $$C_{kr\_s}=\left\{ \begin{array}{ll} \exp\left(-d_{kr\_s}\right), & if\;r\in N_{r}\\ 0, & otherwise \end{array}\right.$$

where $d_{kr\_s}$ is the Euclidean distance between pixels *k* and *r*.

(7) $$C_{kr\_g}=\left\{ \begin{array}{ll} \exp\left(\frac{-{\left\|x_{o}-x_{r}\right\|}^{2}}{\lambda_{g}\cdot \sigma_{kr\_g}^{2}}\right), & if\;r\in N_{r}\\ 0, & otherwise \end{array}\right.$$

where $x_{o}$ is the intensity value of the central reference pixel, and the parameter $\lambda_{g}$ is the gray-level influence factor that controls the degree of influence of adjacent pixels. $\sigma_{kr\_g}=\sqrt{\sum_{r\in N_{r}}{\left\|x_{o}-x_{r}\right\|}^{2}/n_{r}}$ is the deviation of the center pixel $N_{r}$.

(4) The image is filtered using the described local window filter, and the filtering intensity $\xi_{k}$ of the central pixel $x_{k}$ is as in Equation (8).

(8) $$\xi_{k}=\frac{\sum_{r\in N_{k}}C_{kr}\cdot x_{r}}{\sum_{r\in N_{k}}C_{kr}}$$

**Figure 6:**
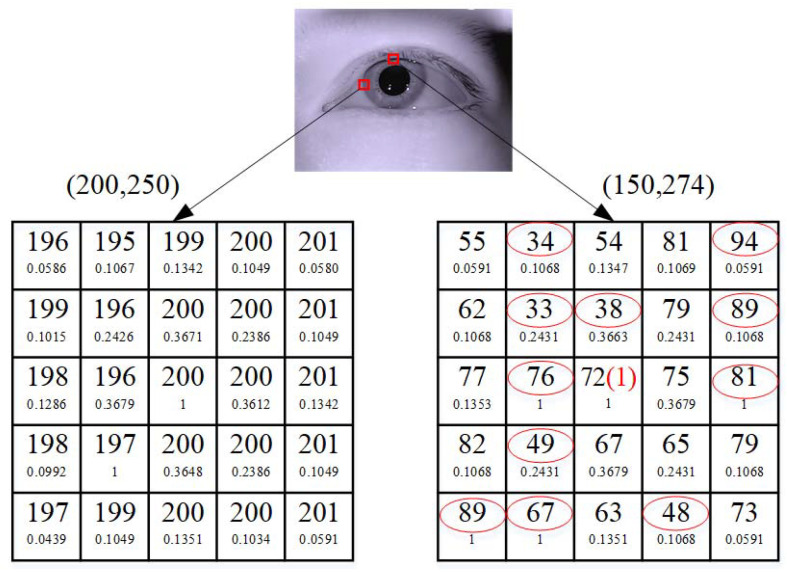
Example of local window filtering.

Two examples of local filtering windows are shown in Figure 6. The upper number in each window unit is the intensity value of each pixel, and the lower number is the weighting coefficient of the local window filter (in this case, the gray level of the influence of adjacent pixels ($\lambda_{g}=3$)). In the example on the left, the intensity value of the pixel at (200, 250) is selected, and the intensity value of the center pixel is not replaced. In the example on the right, the intensity value of the pixel at (150, 274) is selected, and the intensity value of the red circle may be noise or belong to a different category and be evaluated as an unreliable pixel. The original intensity value (72) of the center pixel without obvious noise was replaced by the response intensity value (1) of the local window filter. This example shows that the proposed local window filter is robust to outliers and inhomogeneities.

(5) Calculate the gray histogram of the filtered image to obtain the number of gray levels and the number of pixels with the same gray level.

The adaptive feature extraction of local distribution intensity method is described as follows Algorithm 1:
**Algorithm1:** The adaptive feature extraction of local distribution intensity algorithm.**Input:** grayscale eye image **Output:** filtered eye image(1). Define a local square window $N_{k}$
(2). Calculate the deviation $\sigma_{k}$, evaluate the reliability pixels(3). Calculate the local window weighting coefficient $C_{kr}$
(4). Filter the image

### 2.4. Clustering Segmentation Algorithm Based on Distribution Intensity

Pupil segmentation refers to the classification problem of dividing eye image pixels into pupil pixels and non-pupil pixels. We improve the method of the fuzzy clustering segmentation algorithm based on image distribution information used to detect pupil segmentation in eye images. Use the global distribution information to strengthen the affiliation of the objective function of the fuzzy clustering segmentation algorithm, perform reliable pixel evaluation on the local distribution information of the image, combine the intensity information and the spatial distribution information for filtering, perform clustering to segment the pupil, and obtain a new objective function as in Equation (9).

(9) $$J_{m}=\sum_{i=1}^{C}\sum_{j=1}^{N}\gamma_{j}W_{ij}u_{ij}^{m}{\left\|\xi (x_{j})-v_{i}\right\|}^{2}$$

where $\sum_{i=1}^{C}\sum_{j=1}^{N}\gamma_{j}u_{ij}^{m}{\left\|\xi (x_{j})-v_{i}\right\|}^{2}$ is the objective function of the standard Enhanced FCM [15], $\xi (x_{j})$ represents the pixel $x_{j}$ corresponding to the filtered feature vector, j is the gray value, N represents the number of gray levels in a given eye image, $\gamma_{j}$ represents the number of pixels with gray value equal to *j*, and *C* is the number of specified clusters. The segmentation of the eye image is mainly to achieve the extraction of pupil pixels. Therefore, the pixels are divided into pupil and non-pupil, and $v_{i}$ is the clustering of pupil and non-pupil cluster center, while $u_{ij}^{m}$ represents the membership degree of the pixel belonging to the cluster center $v_{i}$, and *m* represents the ambiguity of the cluster. To solve the optimal classification, by minimizing the objective function, the updated function of membership degree and clustering center can be obtained as in Equations (10) and (11):

(10) $$u_{kj}=\frac{{\left[W_{kj}{}^{\frac{1}{2}}\left(\xi \left(x_{j}\right)-v_{k}\right)\right]}^{-\frac{2}{m-1}}}{\sum_{j=1}^{c}{\left[W_{ij}{}^{\frac{1}{2}}\left(\xi \left(x_{j}\right)-v_{i}\right)\right]}^{-\frac{2}{m-1}}}$$

(11) $$v_{k}=\frac{\sum_{j=1}^{n}\gamma_{j}u_{kj}^{m}W_{kj}\xi \left(x_{j}\right)}{\sum_{j=1}^{n}\gamma_{j}u_{kj}^{m}W_{kj}}$$

The process of the pupil clustering segmentation algorithm based on distribution information is as follows Algorithm 2:
**Algorithm 2:** The process of the pupil clustering segmentation algorithm based on distribution information.**Input**: local neighborhood filtering feature **Output:** cluster center and affiliation matrix(1). Set the maximum number of iterations, the minimum error value, the number of clusters, the fuzzy index, and the value of the neighborhood gray-scale influence factor; (2). Initialize the cluster center and membership matrix; (3). Calculate the affiliation matrix and clustering centers during each iteration using Equations (10) and (11); (4). Judge the iteration stopping condition: if $\left|J_{m}-J_{m-1}\right|\leq e$ or the number of iterations reaches the maximum number of iterations, stop; otherwise, return to step (3).

### 2.5. Postprocessing

When the proposed algorithm converges, the cluster center and the affiliation of each pixel belonging to the cluster center are obtained. In the gray histogram distribution of the eye image, the intensity value of the pupil is the lowest [9], and the smallest component value of the cluster center feature vector is the pupil class center, while the rest of the cluster centers are non-pupil-class centers. The segmented pupil features are used for pupil detection by the method in Reference [9] to locate the pupil centers.

## 3. Experimental Results

### 3.1. Experimental Dataset and Parameter Settings

**Figure 7:**
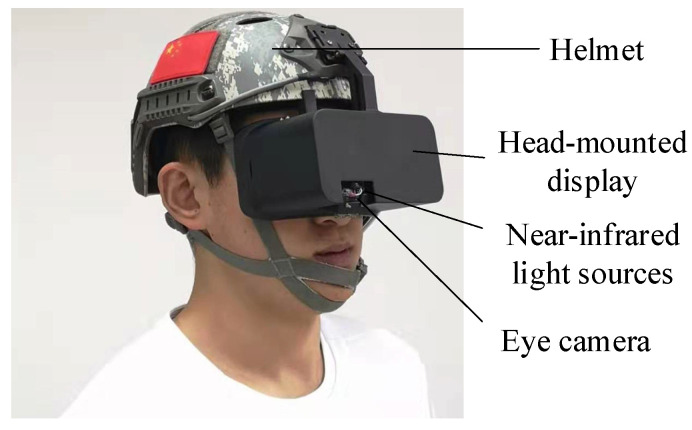
Eye-tracking system device.

The dataset of our research experiment in this paper comes from two parts. Dataset I uses the gaze-tracking system of the research for eye image acquisition (as shown in Figure 7). In Figure 7, our proposed eye-tracking system device is shown, which is a head-mounted eye-tracking system [9], consisting of a helmet, head-mounted display, near-infrared light source, eye camera, and another data-processing module. The eye camera can capture eye images. In our research, we used the Figure 7 device to collect eye images of seven participants continuously gazing at different positions, 1300 images each, in total 9100 images. The acquisition of these eye images is a common step in our usual experimental tests. The experimental conditions have some limitations. Therefore, our own eye map only collects the eye images during continuous gazing, aiming at performing pupil segmentation test on the eye images during movement.

**Figure 8:**
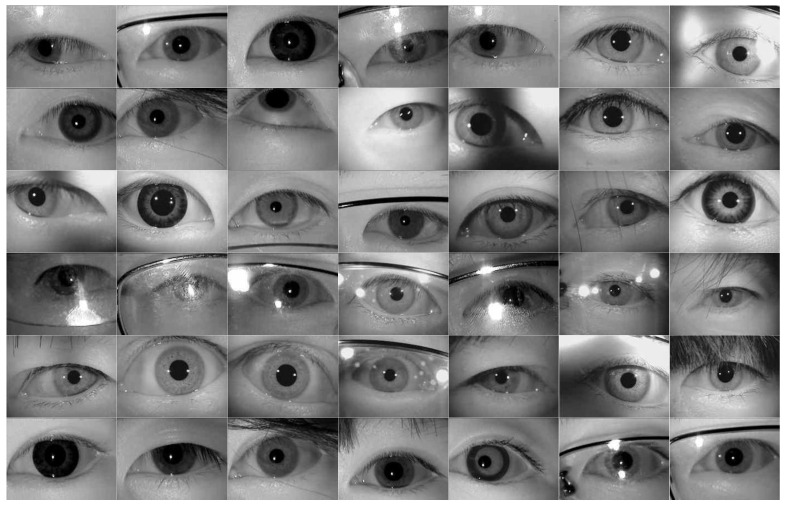
An example of eye images in Dataset II.

Dataset II consists of 500 eye images, selected from the CASIA Iris Dataset [23]. The selected eye images mainly include eye images with obvious pupil characteristics, low pupil contrast, and high-intensity intrusion into the eyes. The CASIA Iris Dataset mainly came from the CASIA Iris Subject Ageing Dataset. In this dataset, for the aging of iris, two different devices were used to collect the old and new eye images of 2009 and 2013 from two periods. There were eye images with glasses, different iris colors, different iris sizes, blurred, and with uneven brightness in order to increase the diversity of eye images in our experiment test. In total, 125 eye images were randomly selected from different folders of the year and equipment under this dataset, and a total of 500 eye images were selected for the experiment test. All the eye images’ sizes are 640 × 480 pixels. An example of eye images in Dataset II is shown in Figure 8. These eye images include different ages, different skin colors, different pupil sizes, wearing glasses or not, eyelash hair invasion, whether there is a shadow in the eye image, different equipment to collect eye image, and other characteristics. Compared with Dataset I, eye image data features are more diverse.

The meanings of the parameters used in our experiment are shown in Table 1.

Three evaluation values of accuracy (AC), sensitivity (SE), and specificity (SP) were used to objectively evaluate the results of pupil segmentation [14,18,19]. We define the evaluation values as in Equation (12).

(12) $$\begin{array}{c} AC=\frac{F_{T}\cap F_{S}}{F_{S}}\\ SE=\frac{F_{T}\cap F_{S}}{F_{T}}\\ SP=\frac{IE-F_{T}\cup F_{S}}{IE-F_{T}} \end{array}$$

where *IE* is the number of pixels of the experimental image, $F_{S}$ is the number of pixels in the segmented pupil region, and $F_{T}$ is the number of pixels in the ground-truth pupil region. Accuracy reflects the ability to accurately segment the pupil; sensitivity refers to the ability to segment the pupil region for the ground-truth pupil; and specificity refers to the ability to correctly determine the pixel points in the non-pupil region. The higher the accuracy, sensitivity, and specificity, the higher quality of accurately segmenting the pupil pixel points.

### 3.2. Dataset I Experiment

**Figure 9:**
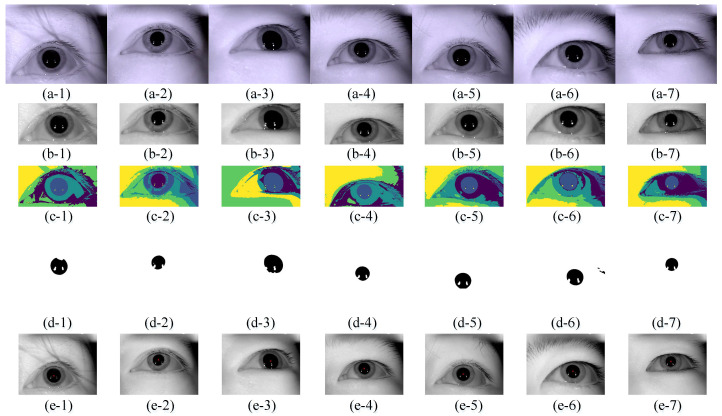
Dataset I image pupil segmentation detection process. (**a-1**)–(**a-7**) are the original eye images of seven different participants; (**b-1**)–(**b-7**) are the eye images after filtration by fusing the image gray intensity information and spatial location information; (**c-1**)–(**c-7**) are the eye images after fuzzy clustering segmentation; (**d-1**)–(**d-7**) are the extracted pupil binarization images; (**e-1**)–(**e-7**) are the detected pupil center locations, and the red cross “×” is the pupil center.

Figure 9 shows the process of pupil segmentation detection of different participants by the proposed method: (a-1)–(a-7) are the original eye images of seven different participants; (b-1)–(b-7) are the eye images after filtration by fusing the image gray intensity information and spatial location information; (c-1)–(c-7) are the eye images after fuzzy clustering segmentation; (d-1)–(d-7) are the extracted pupil binarization images; (e-1)–(e-7) are the detected pupil center locations, and the red dot “×” is the pupil center. Table 2 shows the results of pupil segmentation in the eye images of seven different participants in Figure 9: the second column provides the pupil gray threshold which is from the cluster segmentation result, the third column is the number of iterations using our method, and the fourth column shows pupil location using Reference [9] method.

**Figure 10:**
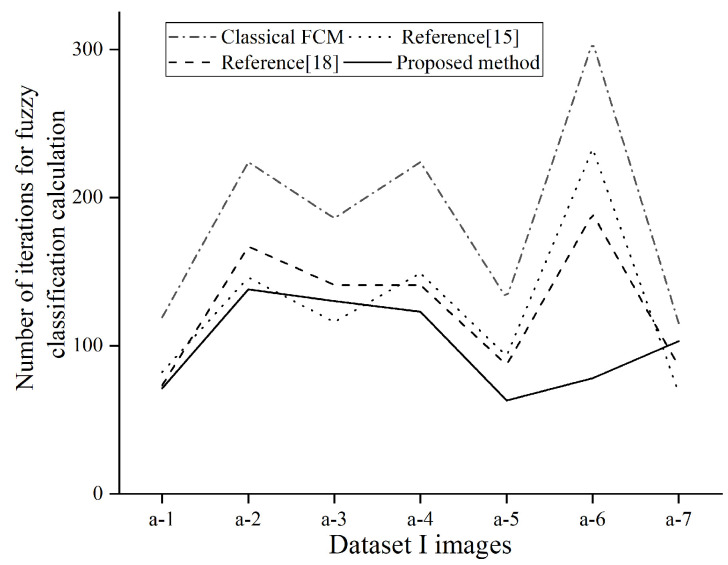
Number of iterations of eye image segmentation.

Figure 10 shows the comparison of the number of iterations of different fuzzy segmentation algorithms for pupil segmentation. The figure shows four fuzzy segmentation algorithms. For the number of iterations of segmentation of the same eye image, the proposed method in this paper is also more time-efficient than the former method.

Table 3 shows the performance comparison of different algorithms for pupil segmentation in Figure 9. Classical FCM, References [15,18], and the proposed method in this paper are fuzzy clustering approach to segmentation. Reference [9] is the threshold segmentation method, which is our work before. The experimental results show that the accuracy and specificity of the proposed method in this paper are better than the segmentation algorithms in the comparative literature, while the sensitivity of Reference [9] is comparatively better. This is probably because of the fact that the proposed method in this paper uses local filtering to characterize the pupil, which responds better to the low-contrast edge part of the features, therefore leading to the subsequent clustering where the low-contrast feature part is accurately considered as a non-pupil class.

Table 4 shows the performance comparison of different algorithms for the average segmentation of eye image pupils in Dataset I. The data show that the segmentation of the pupil of the eye image of Dataset I by the method proposed in this paper has better segmentation quality.

### 3.3. Dataset II Experiment

**Figure 11:**
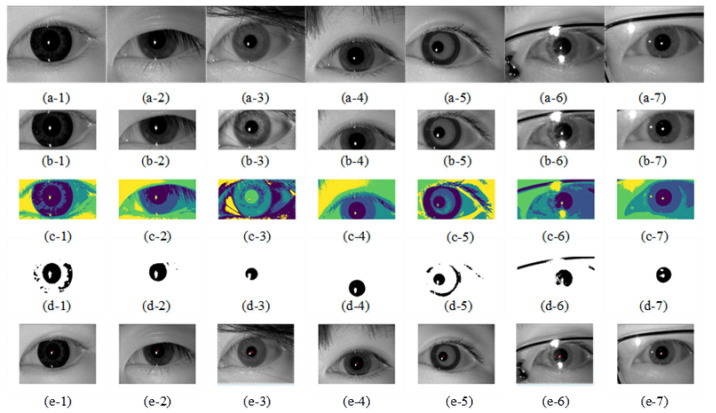
Dataset II image pupil segmentation detection process. (**a-1**)–(**a-7**) are the original images of different low-quality eye images; (**b-1**)–(**b-7**) are the eye images after fusing image gray intensity information and spatial location information filtering; (**c-1**)–(**c-7**) are the eye images after using fuzzy clustering segmentation; (**d-1**)–(**d-7**) are the eye images after extracting the pupil binarization process; (**e-1**)–(**e-7**) are the detected pupil center locations, and the red cross “×” is the pupil center.

Figure 11 shows the pupil segmentation detection process of the proposed method for different low-quality eye images, where (a-1)–(a-7) are the original images of different low-quality eye images; (b-1)–(b-7) are the eye images after fusing image gray intensity information and spatial location information filtering; (c-1)–(c-7) are the eye images after using fuzzy clustering segmentation; (d-1)–(d-7) are the eye images after extracting the pupil binarization process; (e-1)–(e-7) are the detected pupil center locations, and the red cross “×” is the pupil center. Table 5 shows the results of pupil segmentation for different low-quality eye images in Figure 11: the second column provides the pupil gray threshold which is from the cluster segmentation result, the third column is the number of iterations using our method, and the fourth column shows the pupil location using Reference [9] method.

**Figure 12:**
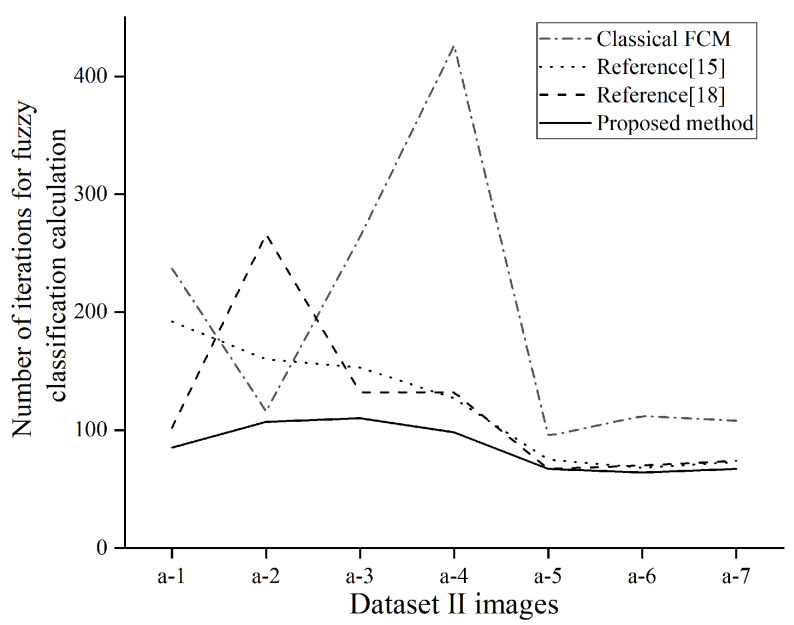
Number of iterations of eye image segmentation.

Figure 12 shows the comparison of the number of iterations for the pupil segmentation. The figure shows four fuzzy segmentation algorithms. For the number of iterations of segmentation of the same eye image, the method proposed in this paper is also more time-efficient than the former method.

Table 6 shows the comparison of the pupil segmentation performance of different algorithms on the eye images of Figure 11. The experimental results show that the accuracy, sensitivity, and specificity of the proposed method in this paper are better than the segmentation algorithms in the comparative literature, and it can segment the pupil with higher quality.

Table 7 shows the comparison of the segmentation result in Dataset II eye images by different methods. The data show the segmentation of the pupil of the eye image of Dataset II by the proposed method in this paper and four other methods. From Table 7, we can see the total accuracy, sensitivity, and specificity of our method which has a better pupil segmentation quality. While the AC value is still low, it might be because the segmentation result has too many non-pupil sections. In the selected eye image, with the reflection of glasses lens and the invasion of similar intensity, in our proposed algorithm, there are still non-pupil feature pixels divided into pupils, and thus, compared with the truly marked pupil region, the segmentation accuracy is low.

On the other hand, although our proposed method had to be verified in two datasets, the pupil segmentation results are relatively better than the same type of fuzzy segmentation algorithm and our group’s previous pupil detection method. In the actual eye-tracking process, locating the pupil and estimating the line of sight is a very complex process with many factors. We put forward that, based on the distribution of divided pupils from the global and local pixel changes, for future research to be more accurate with a variety of eye pupil segmentation, further consideration should be given to various factors in the eye image information to establish a better algorithm to improve pupil segmentation quality.

In summary, the proposed method in this paper to segment pupil features can get a high-quality pupil region. We used four other methods to segment the same eye image. Eye image datasets are made of two parts: one part is our eye images, and the other part is from a public dataset; we only selected 500 images to work with. All of the segmentation results show that our proposed method can get better-quality pupil pixel features, especially, when segmenting low-quality eye images.

## 4. Conclusions

In this paper, we proposed a fuzzy clustering pupil segmentation algorithm based on distribution information. Firstly, preprocessing the eye image, we cropped some non-essential interference factors such as eyebrows, eyelids, and shadows in the eye image, enhanced the contrast between pupils and non-pupil areas, and obtained grayscale eye images. Then, extracting the pupil features, we introduced the global and local distribution information to enhance the membership value and filter images to build a better fuzzy cluster objective function and be able to segment the pupil better. Next, according to the clustering segmentation result, to achieve the pupil threshold and binarization-processing eye image, the pupil characteristics were better highlighted. We could then use the pupil segmentation result to locate the pupil position. The experimental results show that the accuracy, sensitivity, and specificity of our proposed method are better than the existing fuzzy clustering and threshold segmentation methods. Especially for low-quality eye images, including low-contrast and high-intensity features intruding into the eye, as it could segment out the pupil features better. This lays a foundation for accurately detecting the pupil position and contributing to the improvement of the stability and accuracy of the gaze-tracking system.

In future work, we will investigate other possible distribution information processing and fusion methods, such as Dempster–Shafer (D–S) evidence theory, for the distribution information of eye images, so that we can better segment the pupil information in eye images of different quality. We will further investigate how to segment pupils quickly using clustering algorithms and more generally for various low-quality eye image segmentation, improve the speed of segmentation computation, and accurately detect pupils for the line-of-sight estimation.

## Figures and Tables

**Table 1:** Parameters used in the experiment and their meanings.

Symbol	Meaning	Value
*m*	fuzzy factor	2
*C*	number of clusters	5
*t*	maximum number of iterations	1000
*e*	error	0.0001
*w*	local window size	5
*λ_g_*	grayscale impact factor	3

**Table 2:** Pupil segmentation detection results.

Image	Pupil Threshold	Number of Iterations	Pupil Center
a-1	3.90	71	(287.38,330.03)
a-2	3.76	162	(314.50,204.80)
a-3	2.64	130	(399.15,214.77)
a-4	5.64	123	(320.53,272.75)
a-5	3.80	66	(311.72,317.46)
a-6	6.23	78	(379.29,295.15)
a-7	9.82	103	(346.85,217.88)

**Table 3:** Performance comparison of different algorithms.

		Classical FCM	Reference [15]	Reference [18]	Reference [9]	Proposed Method
a-1	AC	63.83%	63.83%	62.73%	50.80%	82.80%
	SE	85.76%	85.76%	86.52%	93.88%	86.80%
	SP	97.63%	97.63%	97.56%	96.56%	100%
a-2	AC	56.84%	55.87%	55.87%	47.48%	81.98%
	SE	85.44%	85.87%	85.87%	89.92%	86.98%
	SP	98.04%	97.99%	97.99%	97.39%	100%
a-3	AC	96.77%	95.85%	95.85%	85.59%	86.39%
	SE	89.25%	89.75%	89.75%	94.75%	90.39%
	SP	99.44%	99.42%	99.42%	99.12%	100%
a-4	AC	60.97%	60.19%	60.19%	52.47%	85.11%
	SE	86.69%	87.19%	87.19%	92.21%	87.11%
	SP	98.22%	98.18%	98.18%	97.74%	100%
a-5	AC	59.64%	58.77%	58.77%	52.13%	86.34%
	SE	88.14%	88.78%	88.78%	93.96%	89.34%
	SP	97.61%	97.57%	97.57%	97.08%	100%
a-6	AC	78.59%	72.01%	72.01%	56.56%	90.78%
	SE	86.33%	87.95%	87.95%	92.19%	88.66%
	SP	98.75%	98.44%	98.44%	97.41%	99.84%
a-7	AC	95.12%	83.61%	83.61%	75.97%	82.63%
	SE	83.57%	85.64%	85.64%	86.69%	86.63%
	SP	99.59%	99.35%	99.35%	99.15%	100%

**Table 4:** Performance comparison of different pupil segmentation algorithms for Dataset I.

	Classical FCM	Reference [15]	Reference [18]	Reference [9]	Proposed Method
AC	73.11%	70.02%	69.86%	60.14%	85.15%
SE	86.45%	86.27%	87.38%	89.94%	87.42%
SP	98.46%	98.36%	98.35%	97.77%	99.98%

**Table 5:** Pupil segmentation detection results.

Image	Pupil Threshold	Number of Iterations	Pupil Center
a-1	4.21	85	(280.67,219.35)
a-2	5.71	107	(323.97,210.65)
a-3	7.93	110	(399.15,214.77)
a-4	2.41	98	(322.04,311.29)
a-5	28.25	67	(285.65,221.44)
a-6	14.51	64	(291.54,134.58)
a-7	3.61	67	(373.05,228.38)

**Table 6:** Performance comparison of different algorithms.

		Classical FCM	Reference [15]	Reference [18]	Reference [9]	Proposed Method
a-1	AC	44.42%	46.96%	42.71%	31.63%	64.94%
	SE	84.58%	84.58%	85.4%	87.68%	90.95%
	SP	94.82%	94.82%	93.93%	90.45%	97.24%
a-2	AC	93.9%	55.85%	51.93%	60.38%	93.93%
	SE	81.11%	87.08%	87.33%	86.86%	89.92%
	SP	99.79%	97.04%	96.57%	97.52%	99.86%
a-3	AC	25.06%	27.56%	24.86%	22.77%	73.95%
	SE	76.22%	76.81%	78.21%	79.85%	80.95%
	SP	95.24%	94.91%	94.21%	93.55%	100%
a-4	AC	98.8%	78.44%	72.02%	94.06%	86.76%
	SE	73.95%	78.8%	78.91%	77.76%	86.62%
	SP	99.96%	99.09%	98.73%	99.73%	100%
a-5	AC	31.71%	27.98%	24.25%	94.07%	32.49%
	SE	80.19%	81.37%	82.39%	85.58%	90.58%
	SP	97.17%	96.12%	95.33%	99.88%	96.83%
a-6	AC	31.85%	88.11%	83.06%	75.05%	40.54%
	SE	31.6%	37.14%	39.55%	40.75%	41.54%
	SP	97.5%	97.17%	97.01%	96.69%	100%
a-7	AC	56.96%	66.14%	64.77%	51.79%	82.35%
	SE	73.79%	75.23%	76.36%	75.11%	82.35%
	SP	98.22%	98.15%	98.09%	97.24%	100%

**Table 7:** The performance of different pupil segmentation algorithms on Dataset II.

	Classical FCM	Reference [15]	Reference [18]	Reference [9]	Proposed Method
AC	55.45%	56.97%	53.22%	61.56%	70.13%
SE	71.59%	74.44%	75.67%	77.19%	79.34%
SP	97.70%	96.77%	96.63%	96.58%	99.07%

## Data Availability

Not applicable.
